# Torsional Characteristics of Carbon Nanotubes: Micropolar Elasticity Models and Molecular Dynamics Simulation

**DOI:** 10.3390/nano11020453

**Published:** 2021-02-11

**Authors:** Razie Izadi, Meral Tuna, Patrizia Trovalusci, Esmaeal Ghavanloo

**Affiliations:** 1 Department of Structural and Geotechnical Engineering, Sapienza University of Rome, Via A. Gramsci, 53, 00197 Rome, Italy; razie.izadi@uniroma1.it; 2 Faculty of Engineering, Yaşar University, Universite Caddesi, Agacli Yol, 37-39, 35100 Izmir, Turkey; meral.tunaeroglu@yasar.edu.tr; 3 School of Mechanical Engineering, Shiraz University, Shiraz 71963-16548, Iran; ghavanloo@shirazu.ac.ir

**Keywords:** micropolar continua, molecular dynamics, SWCNTs parameters identification, optimisation

## Abstract

Efficient application of carbon nanotubes (CNTs) in nano-devices and nano-materials requires comprehensive understanding of their mechanical properties. As observations suggest size dependent behaviour, non-classical theories preserving the memory of body’s internal structure via additional material parameters offer great potential when a continuum modelling is to be preferred. In the present study, micropolar theory of elasticity is adopted due to its peculiar character allowing for incorporation of scale effects through additional kinematic descriptors and work-conjugated stress measures. An optimisation approach is presented to provide unified material parameters for two specific class of single-walled carbon nanotubes (e.g., armchair and zigzag) by minimizing the difference between the apparent shear modulus obtained from molecular dynamics (MD) simulation and micropolar beam model considering both solid and tubular cross-sections. The results clearly reveal that micropolar theory is more suitable compared to internally constraint couple stress theory, due to the essentiality of having skew-symmetric stress and strain measures, as well as to the classical local theory (Cauchy of Grade 1), which cannot accounts for scale effects. To the best of authors’ knowledge, this is the first time that unified material parameters of CNTs are derived through a combined MD-micropolar continuum theory.

## 1. Introduction

Discovered by Iijima [1,2], carbon nanotubes (CNTs) have received great attention due to their superior mechanical, optical, thermal and electrical properties [3,4,5,6,7]. Their exhaustive application in nanoscopic field as springs [8], oscillators [9,10], actuators [11,12], transistors [12], sensors [13] and reinforcement element [14,15,16,17], requires a complete understanding of the underlying mechanical properties. In doing so, two main approaches, namely discrete and multiscale continuum models are favored depending on the specificity of the problem, while experiments are not preferred due to the design complexity and expenses in nano-scale.

In discrete modelling technique, atoms and interatomic bonds are explicitly modelled using first-principle or semi-empirical methods (e.g., tight binding, ab initio models, molecular dynamics (MD) simulation) [18,19,20]. Despite their capability of representing the actual behaviour of the media, discrete modelling techniques are not very practical due to their computational expense, which rapidly increase with total number of degree of freedoms [21]. Classical theory of elasticity (Cauchy of Grade 1), on the other hand, fails to accurately homogenize the discrete nature into a continuum medium for a structure having comparable internal and external lengths [22,23]. This drawback can be overcome by means of non-classical (non-local) continuum theories which simultaneously utilize field description at coarse level, and preserve the memory of material’s underlying structure at fine level through internal scale parameters [24,25,26,27,28,29] that can refer to different physical features ranging from nano order (e.g., distance between atoms in a nanoscopic structure) up to meso/macro orders (size of particle/grain in a composite medium) as demonstrated in different studies [30,31,32,33].

Incorporation of non-locality related parameters to the continuum theory might be obtained through additional kinematic descriptors (non-standard primal fields) and work-conjugated stress measures (non-standard dual fields) as in ‘implicit/weak’ non-local models (multi-field continua) or through constitutive equations containing integral, integro-differential or finite-difference operators of spatial fields as in ‘explicit/strong’ non-local models [22,29,34,35]. Although the majority of literature, that deals with continuum modelling of CNTs [36,37,38,39,40,41], employs explicitly non-local Eringen’s theory of elasticity, as it perfectly captures the long-range interaction between atoms by relating stress at a point to the strain of entire domain through an attenuation-type kernel function [42], few works are devoted to the application of implicitly non-local microcontinuum field theories. For instance, Xie and Long [43] adopted micropolar theory to calculate the fundamental frequencies of single-walled CNTs (SWCNTs) and double-walled CNTs (DWCNTs), while others applied couple stress continuum theory (i.e., a subcase of micropolar theory with an internal constraint; micro-rotations equal the macro-rotation [44,45,46]) [47,48,49]. Akgoz and Civalek [47] calculated the critical axial compressive buckling loads of CNTs using the Euler-Bernoulli beam model based on the modified couple stress theory, and presented the influence of length scale parameter on the buckling characteristics. Khajueenejad and Ghanbari [48] studied axial buckling of CNTs using Timoshenko beam model and modified couple stress theory. By correlating the derived buckling loads with the MD simulations data of Liew et al. [50], they estimated the variable couple stress internal length parameter for zigzag and armchair nanotubes. Akbarzadeh and Soltani [49] added another length scale parameter to the modified couple stress theory to match the theoretical relation for longitudinal and flexural dispersion of CNTs to the data provided by Wang et al. [51].

In the light of these informations, micropolar continuum theory is deemed as a good candidate to model size dependent mechanical behaviour of CNTs as underlying hexagonal lattice structure is consistent to the essence/existence of micro-rotations, hence encourages utilization of theories endowed with additional kinematic descriptors [29,52,53,54]. To determine corresponding material parameters, a wide range of armchair and zigzag nanotubes are examined by both discrete and continuum modelling techniques. In doing so, primarily, MD simulations of nanotubes having different diameters but same aspect ratio are performed to show the occurrence of size effects through interpreting the torsional data (i.e., torsional rigidity) [55,56]. Since herein the focus is on torsional rigidities, which is shown to be independent of the aspect ratio of corresponding nanotube [38], moderately short nanotubes are studied for computational efficiency. Note that different states of loading exhibiting size effects (e.g., bending) are possible. Although all would yield same material parameters, imposing the end conditions for torsional loading in MD simulations is much more straightforward and accurate compared to other types of loading. For continuum modelling, CNTs are considered as micropolar beams whose cross-sectional area remain planar after deformation (i.e., no warping), and according closed-form expressions of apparent shear modulus are re-derived considering both solid and hollow cross-sections. Even though CNTs genuinely are hollow cylinders, solid cross-section case can be an expedient while dealing with continuum modelling of nanotubes employed as reinforcement elements [57,58,59,60]. Finally, a unified parameter set, as well as thickness, of equivalent continuum model is derived for each class of SWCNTs chirality by comparing the corresponding data obtained from discrete MD and continuum models (micropolar, couple stress and classical) by virtue of a non-linear optimisation approach. To highlight the importance of having skew-symmetric stress and strain measures as in micropolar theory, optimisation process is re-performed by defining an internal constraint on micro-rotations leading to symmetric couple stress theory. To the best of authors’ knowledge this is the first time that a combined MD-micropolar continuum theory is employed to model the size-dependent mechanical behaviour of CNTs.

The rest of the paper is organised as follows; Section 2 describes the details on MD simulation and presents the primary obtained results. In Section 3, the governing equations and the derivation of relations of micropolar torsional rigidities are presented. In Section 4, the non-local material parameters are determined comparing the results of MD simulation and continuum model through a non-linear optimisation approach. Finally, the concluding remarks are presented in Section 5.

## 2. Discrete Model

**Figure 1:**
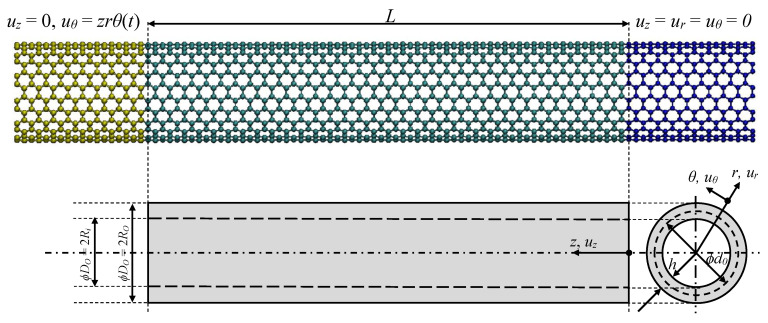
Schematic illustration of SWCNT and its equivalent continuum model boundary conditions for torsional loading.

Single-walled carbon nanotubes (SWCNTs) can be described as a single layer of graphene sheet that has been rolled into a seamless hollow cylinder with a constant radius. They have a mean diameter, $d_{0}$, a total length, *L* (see Figure 1), and further categorised as armchair (m=n), zigzag (n=0) and chiral (m≠n) according to the direction of wrapping:

(1) $$C_{h}=ma_{1}+na_{2}=\left(m,n\right)$$

where $C_{h}$ refers to chiral vector, and is specified by chiral index (m,n), alongside with basis vectors of graphene lattice $a_{1}$ and $a_{2}$ [61,62,63,64]. For the sake of computational cost, the analyses herein are performed considering eight armchair and eight zigzag nanotubes having different mean diameters $d_{0}=6.3-16.3\;Å$ ($1\;Å=10^{-10}\;m$), while overall lengths are selected to result in moderately short nanotubes; $L\approx 5.0\;d_{0}$, since torsional stiffness is proven to be independent of the aspect ratio through MD simulations performed for (5,5) nanotubes having different lengths, as also reported in the literature [38,65]. The discrete models are built using Visual Molecular Dynamics (VMD) software developed by University of Illinois [66], and corresponding MD simulations are carried out by employing the open source Large-scale Atomic/Molecular Massively Parallel Simulator (LAMMPS) package, that is widely used for ensembles of solid, liquid or gaseous particles [67].

MD simulation has been introduced by Alder and Wainwright [68,69], as a computational method that allows one to predict the time evolution of atomic/molecular systems, based on classical Newtonian mechanics, and generally admitted as *numerical* or *computer* experiments. In MD, atoms are assumed as interacting solid spheres, the position, r, velocity, v, and acceleration, a, of each atom are to be determined for each time step, *t*, via solving corresponding differential equations system, by virtue of integration algorithms [70]:

(2) $$F_{i}=-\nabla_{r_{i}}U\left(r_{1},\ldots ,r_{N}\right)=m_{i}\frac{d^{2}r_{i}\left(t\right)}{dt^{2}}$$

where F refers to the force vector acting on the *i*-th atom, and can be written as the gradient of the total potential energy function, *U*, of a system consisting of *N* atoms. In the most general case, energy function includes one-body, $U_{1}$, (describes external force fields or boundary conditions), two-body, $U_{2}$, (depends on the distance between pairs of atoms) and three-body (or higher-order), $U_{3}$, (based on the geometry of the atomic arrangement) potentials:

(3) $$U\left(r_{1},\ldots r_{N}\right)=\sum_{i}U_{1}\left(r_{i}\right)+\sum_{i}\sum_{j\neq i}U_{2}\left(r_{i},r_{j}\right)+\sum_{i}\sum_{j\neq i}\sum_{k\neq i,j}U_{3}\left(r_{i},r_{j},r_{k}\right)+\ldots$$

In the present study, simulations are performed in a canonical (NVT) ensemble where the total number of atoms, volume of the simulation box, and the temperature of the system are kept constant [67]. The velocity-Verlet integrator algorithm is used with a time step of 1 fs (1 femtosecond $=1\times 10^{-15}$ s), and the interatomic relation between carbon atoms is described using adaptive intermolecular reactive empirical bond order (AIREBO) potential with a cut-off radius of 10.2 Å [71]. The temperature is kept constant at 10 Kelvin (K) to reduce noise and thermal effect with the aid of Nose-Hoover thermostat, while before the thermalisation, an energy minimization is conducted by using the conjugate gradient algorithm in order to remove the primitive forces above $1\times 10^{-12}$ eV/A. After thermalisation, clamped-free nanotubes are subjected to torsional loading by a sequential rotation and relaxation route, called as displacement-rate controlled simulation. To apply the corresponding boundary conditions, movement of atoms located at the right end are restricted in all directions, while axially fixed atoms at the left end are twisted along *z*-axis with an incremental twist angle of 0.006 radians (see Figure 1). After each rotation, the remaining atoms (those that are not fixed) are subjected to NVT ensemble for 20,000 time steps (20 picosecond = $20\times 10^{-12}$ s) to let the structure attain the minimum energy state at each increment, which corresponds to a twist angle speed of 0.0003 rad/ps, and ensures quasi-static state. The span of fixed atoms at each end, $l_{\mathrm{fixed}}$, is arranged to be in accordance with the overall length of the nanotube, such that the ratio in-between remains constant (i.e., $l_{\mathrm{fixed}}/L\approx 25\%$).

During simulation, the total potential energy, *U*, torsional moment, *T*, and twist angle at the tip, θ, are recorded at the end of each increment (i.e., every 20 ps) by averaging the last twenty data. Corresponding torsional stiffness, *K*, which is derived as follows, are tabulated in Table 1 alongside with critical buckling angles, $\theta_{crt}$,

(4) $$\begin{array}{c} K=\frac{\partial^{2}U}{\partial \theta^{2}} \end{array}$$

**Figure 2:**
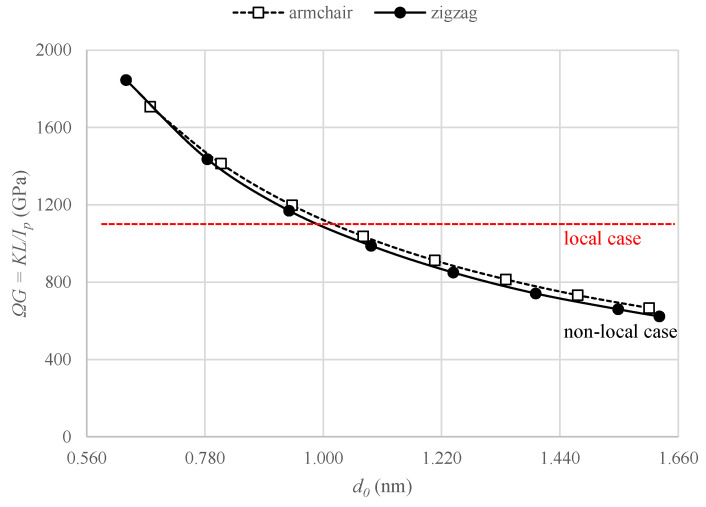
Variation of apparent shear modulus with respect to mean diameter for armchair and zigzag CNTs, considering solid cross section.

As reported by Gauthier and Jahsman [55], the apparent shear modulus, $\left(\Omega G=KL/I_{p}\right)$, in torsional loading of a cylindrical specimen with micropolar non-local character increases with decreasing diameter. This is in contradiction to the local case for which no size variation is expected; indeed, the apparent shear modulus, $J/I_{p}$, turns out to be a constant, *G*, and independent of the diameter of the specimen (Figure 2). To check if the non-local behaviour is verified for nanotubes considered herein, the variation of apparent shear modulus, which is calculated using the torsional stiffness values obtained from MD simulations, with respect to diameters is plotted at Figure 2, as a preliminary investigation. Even though the thickness of nanotubes is not known yet, polar moment of inertia can be obtained by assuming the case of solid cross section; $I_{p}=\left(\pi d_{0}^{4}\right)/32$. The trend of curve evidently verifies the non-local behaviour since apparent shear modulus depends on the diameter as opposed to what is observed in local case.

The reaction forces emerged at each end are documented to assure that no significant axial force, which might spoil the pure torsion character of loading, is generated. By increasing the relaxation time from 5000 to 60,000 fs (i.e., 5000, 10,000, 20,000, 60,000 fs) while keeping incremental twist angle fixed to 0.006 rad, the effect of twist rate is studied through comparing torsional rigidity of carbon nanotubes having a chirality of (12,12), which requires the longest relaxation time to find the equilibrium position of atoms among the models considered herein.

For validation of MD simulations, torsional rigidity ($J=KL=T_{crt}L/\theta_{crt}$), which is considered as the main measured quantity in torsional testing, the data of clamped-free armchair and zigzag nanotubes with (10,10) and (16,0) chirality are compared with those of Chowdhurry [65]. Corresponding relative difference between the results, which are 1.28%, 2.03% respectively, proves the accuracy of the simulations, and the independence of the torsional stiffness from the total length of the nanotube.

## 3. Equivalent Continuum Models

In the present section, the governing equations for the pure torsion of a circular cylinder made of homogeneous isotropic micropolar elastic material is presented. Then, the steps to the derivation of micropolar torsional rigidities are outlined. All the equations are performed within the linearised kinematic framework. CNT is modelled as a micropolar continuum beam, with considering both cases of solid and hollow cross sections.

### 3.1. Micropolar Hollow Circular Cylinder

In this section, the solution strategy is based on Taliercio’s study on the micropolar hollow cylinder under twist [72]. Figure 1 shows the equivalent circular cylinder of length *L* in a cylindrical coordinate system (r,θ,z) with *z* aligned with cylinder axis. $R_{o}$ and $R_{i}$ denote the outer and inner radii, respectively.

While fixing one end of the cylinder (z=0), the other end (z=L) is subjected to a rotation $\theta_{t}\;L$ about the cylinder axis leading to the following displacement and microrotation fields:

(5) $$u_{r}=0,\;u_{\theta }=\theta_{t}rz,\;u_{z}=0,\;\phi_{r}=\theta_{t}\Phi \left(r\right),\;\phi_{\theta }=0,\;\phi_{z}=\theta_{t}z$$

where $\theta_{t}$ is the unit angle of twist, and $u_{i}$ and $\phi_{i}$ (i=r,θ,z) denote the displacement and microrotation components, respectively. In Equation (5), Φ(r) is an unknown material and geometrical function with length dimension, which denotes the coupling between micro-rotations in axial and radial directions. The validation of the assumption in Equation (5) is guaranteed as the final solution which fulfills all the equations will be found. According to Kirchhoff’s theorem in linear elasticity this must be the exact solution. In accordance with displacement and microrotation fields given in Equation (5), no warping occurs during deformation and the cross section remains planar.

In a micropolar body, both the position and orientation are used to describe the material particles [26,28,52,73]. Under these assumptions the kinematic compatibility equations are:

(6) $$\varepsilon_{ij}=u_{j,i}+e_{jik}\phi_{k},\;\chi_{ij}=\phi_{j,i}\;\left(i,j=1,2,3\right)$$

where $\varepsilon_{ij}$ and $\chi_{ij}$ denote the components of micropolar strain and curvature tensors and $\epsilon_{ijk}$ is the third order permutation tensor. Substituting Equation (5) into Equation (6), the only non-zero components become:

(7) $$\varepsilon_{\theta z}=-\theta_{t}\Phi \left(r\right),\;\;\;\varepsilon_{z\theta }=\theta_{t}\left(r+\Phi \left(r\right)\right),\;\;\;\chi_{rr}=\theta_{t}\Phi^{\prime }\left(r\right),\;\;\;\chi_{\theta \theta }=\theta_{t}\frac{\Phi (r)}{r},\;\;\;\chi_{zz}=\theta_{t}$$

where the prime denotes derivative with respect to *r*.

The constitutive equations for a linear isotropic micropolar material read:

(8) $$\sigma_{ij}=\lambda \varepsilon_{kk}\delta ij+\mu \varepsilon_{ji}+\left(\mu +\kappa \right)\varepsilon_{ij},\;\mu_{ij}=\alpha \chi_{kk}\delta ij+\beta \chi_{ji}+\gamma \chi_{ij}$$

Equation (8) contains six elastic material constants; the two Lamé’s constants, λ, and μ and four additional parameters, α, β, γ and κ emerging from micropolar theory [56]. The following so-called engineering constants are also derived based on the aforementioned six parameters providing better physical insight [56]:

- G=μ+κ/2: shear modulus
- $N=\sqrt{\kappa /(2(\mu +\kappa ))}$: coupling number
- $l_{t}=\sqrt{\left(\beta +\gamma )/(2\mu +\kappa \right)}$: charachteristic lenght for torsion
- ψ=(β+γ)/(α+β+γ): polar ratio
- E=(2μ+κ)(3λ+2μ+κ)/(2λ+2μ+κ): Young’s modulus
- ν=λ/(2λ+2μ+κ): Poisson’s ratio
- $l_{b}=\sqrt{\gamma /2(2\mu +\kappa )}$: charachteristic lenght for bending

Substituting the strains and curvatures in Equation (6) into the constitutive law (Equation (8)) leads to the following stress and couple-stresses relations:

(9) $$\begin{array}{cc} \sigma_{\theta z} & =\left(\mu r-\kappa \Phi \left(r\right)\right)\theta_{t}\;\sigma_{z\theta }=\left(\left(\mu +\kappa \right)r+\kappa \Phi \left(r\right)\right)\theta_{t}\\ \mu_{rr} & =\left[\left(\alpha +\beta +\gamma \right)\Phi^{\prime }\left(r\right)+\alpha \left(\frac{\Phi (r)}{r}+1\right)\right]\theta_{t}\\ \mu_{zz} & =\left[\left(\alpha +\beta +\gamma \right)+\alpha \left(\Phi^{\prime }\left(r\right)+\frac{\Phi (r)}{r}\right)\right]\theta_{t}\\ \mu_{\theta \theta } & =\left[\left(\alpha +\beta +\gamma \right)\frac{\Phi (r)}{r}+\alpha \left(\Phi^{\prime }\left(r\right)+1\right)\right]\theta_{t} \end{array}$$

In the absence of body force and body couples, the equilibrium equations can be derived in the form:

(10) $$\sigma_{ji,j}=0,\;\mu_{jk,j}-e_{ijk}\sigma_{ji}=0\;\left(i,j=1,2,3\right)$$

As the non-vanishing non-symmetric stress and couple stress measures expressed in Equation (9) are independent of θ and *z*, only the following relation corresponding to angular equilibrium equation in radial direction remains:

(11) $$\frac{\partial \mu_{rr}}{\partial r}+\frac{\mu_{rr}}{r}+\sigma_{\theta z}-\sigma_{z\theta }-\frac{\mu_{\theta \theta }}{r}=0$$

It results in the following differential equation for Φ(r):

(12) $$r^{2}\Phi^{\prime \prime }\left(r\right)+r\Phi^{\prime }\left(r\right)-\left(1+p^{2}r^{2}\right)\Phi \left(r\right)-\frac{p^{2}}{2}r^{3}=0$$

which has the solution:

(13) $$\Phi \left(r\right)=AI_{1}\left(pr\right)+BK_{1}\left(pr\right)-r/2$$

where $p^{2}=2k/\left(\alpha +\beta +\gamma \right)$ and $I_{n}$ and $K_{n}$ are the modified Bessel functions of the first and second kind of order *n*.

From balance considerations, the following relations hold as the boundary conditions:

(14) $$t_{i}=\sigma_{ji}n_{j},\;\mu_{i}=\mu_{ji}n_{j}\;\left(i,j=1,2,3\right)$$

where $t_{i}$ and $\mu_{i}$ are the components of surface traction and surface couple traction, respectively and $n_{j}$ is the unit outward normal vector of the continuum boundary. The coefficient *A* and *B* are determined by applying traction free boundary conditions on the lateral surfaces ($r=R_{i}$ and $r=R_{o}$):

(15) $$\begin{array}{cc} A & =\frac{2R_{o}R_{i}s\kappa \left(K_{0}\left(pR_{i}\right)-K_{0}\left(pR_{o}\right)-s^{2}\left(R_{i}K_{1}\left(pR_{o}\right)-R_{o}K_{1}\left(pR_{i}\right)\right)\right)}{▵}\\ B & =\frac{2R_{o}R_{i}s\kappa \left(I_{0}\left(pR_{i}\right)-I_{0}\left(pR_{o}\right)+s^{2}\left(R_{i}K_{1}\left(pR_{o}\right)-R_{o}I_{1}\left(pR_{i}\right)\right)\right)}{▵} \end{array}$$

with s=p(β+γ) and

(16) $$\begin{array}{cc} ▵ & =2\left(2R_{o}\kappa I_{0}\left(pR_{o}\right)-sI_{1}\left(pR_{o}\right)\right)\left(2R_{i}\kappa K_{0}\left(pR_{i}\right)+sK_{1}\left(pR_{i}\right)\right)\\ \; & -2\left(2R_{i}\kappa I_{0}\left(pR_{i}\right)-sI_{1}\left(pR_{i}\right)\right)\left(2R_{i}\kappa K_{0}\left(pR_{o}\right)+sK_{1}\left(pR_{o}\right)\right) \end{array}$$

The torsional rigidity of the micropolar cylinder, $J_{m}$, is defined as the ratio of the applied twisting torque (*T*) on any cross section (*C*) to the unit angle of twist:

(17) $$J_{m}=\frac{T}{\theta_{t}}=\frac{\int_{C}\left(\sigma_{z\theta }r+\mu_{zz}\right)\;dC}{\theta_{t}}$$

In Equation (17), $\sigma_{z\theta }$ and $\mu_{zz}$ can be found by substituting Φ(r) from Equation (13) into the related expressions in Equation (9) as follows:

(18) $$\begin{array}{cc} \sigma_{z\theta } & =\left(\left(\mu +\kappa \right)r+\kappa \left(AI_{1}\left(pr\right)+BK_{1}\left(pr\right)-r/2\right)\right)\theta_{t}=\left(Gr+\kappa f_{1}\right)\theta_{t}\\ \mu_{zz} & =[\left(\alpha +\beta +\gamma \right)+\alpha (A\left(pI_{0}-\frac{I_{1}}{r}\right)+B\left(-pK_{0}-\frac{K_{1}}{r}\right)-\frac{1}{2}+\ldots \\ \; & \frac{1}{r}\left(AI_{1}\left(pr\right)+BK_{1}\left(pr\right)\right))]\theta_{t}\\ \; & =\left[\left(\beta +\gamma \right)+\alpha pf_{0}\right]\theta_{t} \end{array}$$

where

(19) $$\begin{array}{cc} f_{0} & =AI_{0}\left(pr\right)-BK_{0}\left(pr\right)\\ f_{1} & =AI_{1}\left(pr\right)+BK_{1}\left(pr\right) \end{array}$$

Using integral relations for modified Bessel functions [74] and integrating over the cross section (C), one can obtain:

(20) $$\begin{array}{cc} J_{m} & =G\left(\pi \frac{R_{o}^{4}-R_{i}^{4}}{2}\right)+\left(\beta +\gamma \right)\left(\pi \left(R_{o}^{2}-R_{i}^{2}\right)\right)+\ldots \\ \; & 2\pi \left[\frac{A}{p}\left(p\alpha \left(rI_{1}\left(pr\right)\right)+\kappa r^{2}I_{2}\left(pr\right)\right)+\right.\ldots \\ \; & B{\left.(\alpha \left(rK_{1}\left(pr\right)\right)-\frac{\kappa r^{2}}{p}K_{2}\left(pr\right))\right]}_{R_{i}}^{R_{o}} \end{array}$$

A more useful quantity to define is the nondimensional rigidity ratio for hollow cylinders $\Omega_{h}$, which is the ratio of micropolar torsional rigidity to the classical counterpart:

(21) $$\Omega_{h}=\frac{J_{m}}{J_{ch}}=1+\frac{{\left(2l_{t}\right)}^{2}}{R_{o}^{2}-R_{i}^{2}}+\frac{4A\left(p\alpha \eta_{1}^{I}+\kappa \eta_{2}^{I}\right)+4B\left(p\alpha \eta_{1}^{II}-\kappa \eta_{2}^{II}\right)}{pG(R_{o}^{4}-R_{i}^{4})}$$

where $J_{ch}=G\left(\pi \frac{R_{o}^{4}-R_{i}^{4}}{2}\right)$ is the well-known classical torsional rigidity for hollow cylinders, where $\eta_{n}^{I}=R_{o}^{n}I_{n}\left(pR_{o}\right)-R_{i}^{n}I_{n}\left(pR_{i}\right)$ and $\eta_{n}^{II}=R_{o}^{n}K_{n}\left(pR_{o}\right)-R_{i}^{n}K_{n}\left(pR_{i}\right)$, n=1,2.

It should be noted that, although, the solution method in this section is mainly based on [72], the final formulation is different and more convenient to adopt. A new parameter $\eta_{2}^{II}$ is defined, instead of the parameter named ξ in [72], avoiding the uneasy calculation of generalized Meijer G function, $G_{pq}^{mn}$, in ξ evaluation;

(22) $$\xi =R_{o}^{3}G_{31}^{21}\left(\frac{pR_{o}}{2},\frac{1}{2}\left|{}_{\frac{-1}{2}\frac{1}{2}\frac{-3}{2}}^{\frac{-1}{2}}\right.\right)-R_{i}^{3}G_{31}^{21}\left(\frac{pR_{i}}{2},\frac{1}{2}\left|{}_{\frac{-1}{2}\frac{1}{2}\frac{-3}{2}}^{\frac{-1}{2}}\right.\right)$$

### 3.2. Micropolar Solid Circular Cylinder

In an early study conducted by Gauthier and Jahsman [55], the following relation is proposed for the torsional rigidity ratio of a micropolar solid cylinder of radius *R*:

(23) $$\Omega_{s}=\frac{J_{m}}{J_{cs}}=1+6{\left(\frac{l_{t}}{R}\right)}^{2}\frac{1-\frac{4}{3}\psi X}{1-\psi X}$$

where $X=(I_{1}\left(pR\right)/pRI_{0}\left(pR\right))$ and $J_{cs}=G\left(\pi \frac{R^{4}}{2}\right)$ denotes the classical torsional rigidity for solid cylinders.

An alternative expression can be easily obtained by considering $R_{i}=0$, in the case of hollow cylinder introduced in the previous section. In order to avoid singularity at the origin, the boundary conditions change to Φ(0)=0 and $\mu_{rr}\left(R_{o}\right)=0$, leading to the following coefficient constants [72]:

(24) $$A=\frac{Rs}{2(2R\kappa I_{0}\left(pR\right)-sI_{1}\left(pR\right))},\;B=0$$

The obtained rigidity ratio through the two equations (Equations (23) and (24)) can be proved to be equal.

**Figure 3:**
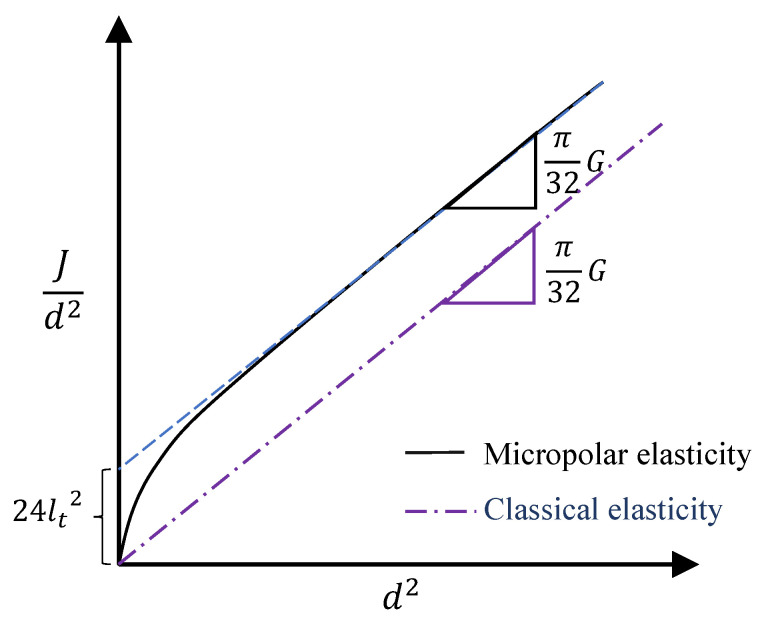
Plot of torsional rigidity divided by the square of the diameter vs. the square of the external diameter to characterize Cosserat parameters.

Based on the relation in Equation (23), Lake [56] proposed to plot torsional rigidity divided by the square of the diameter vs. the square of the diameter to characterize Cosserat parameters (see Figure 3); the slope of the asymptotic straight line is proportional to the shear modulus, *G*, and the characteristic lengths, $l_{t}$, can be extracted from the intercept of the extrapolated straight portion of the curve upon the ordinate. The coupling number *N* and polar ratio ψ also affect the plot shape, especially at the vicinity of the origin, however, they cannot be precisely determined solely using the plot.

Finally, it should be noted that according to both Equations (21) and (23), for micropolar bodies, $\Omega_{h}\;\mathrm{and}\;\Omega_{s}\geq 1$. This means that the torsional rigidity is higher that expected classically. This reveals the occurrence of “size effect” [56] which is more dominant in smaller diameters, where the internal length becomes comparable to cylinder diameter.

In addition, let us name the product $\Omega_{h}G$ or $\Omega_{s}G$ as the apparent shear moduli as suggested by [55]. This definition is expedient as in a pure torsion experiment, it can be easily calculated through torque-twist measurements:

(25) $$\Omega_{h}G=\frac{T}{\theta_{t}\pi \left(R_{o}^{4}-R_{i}^{4}\right)/2}\;\mathrm{or},\;\Omega_{s}G=\frac{T}{\theta_{t}\pi \left(R^{4}\right)/2}$$

It should be noted that if $\Omega_{h}$ or $\Omega_{s}$ are equal to unity, the classical Cauchy relation for shear modulus is recovered as follows;

(26) $$G=\frac{T}{\theta_{t}\pi \left(R_{o}^{4}-R_{i}^{4}\right)/2}\;\mathrm{or},\;G=\frac{T}{\theta_{t}\pi \left(R^{4}\right)/2}$$

Finally, considering the mean diameter, $d_{0}$, and then substituting into Equations (21) and (23), the expressions for apparent shear moduli can be written as follow:

(27) $$\begin{array}{cc} \Omega_{h}G & =G\left(1+\frac{{\left(4l_{t}\right)}^{2}}{{\left(d_{0}+h\right)}^{2}-{\left(d_{0}-h\right)}^{2}}\right)+\frac{64A\left(p\alpha \eta_{1}^{I}+\kappa \eta_{2}^{I}\right)+64B\left(p\alpha \eta_{1}^{II}-\kappa \eta_{2}^{II}\right)}{p({\left(d_{0}+h\right)}^{4}-{\left(d_{0}-h\right)}^{4})}\\ \Omega_{s}G & =G(1+6{\left(\frac{2l_{t}}{d_{0}}\right)}^{2}\frac{1-\frac{4}{3}\psi X}{1-\psi X}) \end{array}$$

These equations are expressed in terms of micropolar parameters and, in the case of hollow cylinders, also in terms of thickness, *h*.

### 3.3. Couple Stress Theory

For homogenization of discrete structures possessing strong anisotropy as orthotropic textures, a full micropolar model is needed (for more discussion on the topic see [31,54,75]). In this case, the non-symmetries (skew-symmetries) of the strain and stress tensors play a relevant role. However, if the material texture exhibits special groups of symmetries, as orthotetragonal textures, the non-symmetries of the stresses and strains disappear. In this case, the couple stress theory may be considered as a good candidate (in a different framework see for instance [76]). For a chiral CNT in general, the non-symmetries of strain and stress in the axial and circumferential directions are quite predictable, therefore, a full micropolar model is to be preferred for the homogenization process. However, the symmetrical structure of armchair and zigzag CNTs, triggers the possibility of the application of couple stress model with lower number of unknown material parameters to be characterized. This will also be examined in the present paper.

In couple stress theory, the micro-rotations are constrained to follow the local rigid rotation:

(28) $$\phi_{i}=-\frac{1}{2}\epsilon_{ijk}u_{j,k}$$

that leads to the classical compatibility equation;

(29) $$\varepsilon_{ij}=\frac{1}{2}\left(u_{i,j}+u_{j,i}\right)$$

Herein, couple stress theory is attained by imposing the symmetries on the strain tensor, hence, according to Equation (7), the only non-zero strain terms in the pure torsion problem are found to be $\varepsilon_{\theta z}$ and $\varepsilon_{\theta z}$, and their equivalency results in:

(30) $$\Phi \left(r\right)=-\frac{r}{2}$$

By substituting Equation (30) into Equation (9), one can obtain the following relations for the stresses and couple-stresses;

(31) $$\begin{array}{cc} \sigma_{z\theta } & =\sigma_{\theta z}=\left(\mu +\frac{\kappa }{2}\right)r\theta_{t}=Gr\theta_{t}\\ \mu_{zz} & =\left(\beta +\gamma \right)\theta_{t}=2Gl_{t}^{2}\theta_{t}\\ \mu_{\theta \theta } & =\mu_{rr}=-\frac{\left(\beta +\gamma \right)}{2}\theta_{t}=-Gl_{t}^{2}\theta_{t} \end{array}$$

Note that the obtained strains, rotations, stresses and couple stresses are quite consistent with the torsion problem of a circular cylinder, proposed by Yang et al. [77] in the development of a modified couple stress theory, except that in their study the relations are presented in terms of deviatoric part of the couple stress tensor, $m_{ij}$, while in the present problem $\mu_{ij}=m_{ij}$, as $\mu_{ii}=0$.

Finally, division of the applied torque to the unit angle of twist gives the torsional rigidity for the couple stress model, $J_{co}$:

(32) $$\begin{array}{cc} J_{co} & =\frac{T}{\theta_{t}}=\frac{\int_{C}\left(\sigma_{z\theta }r+\mu_{zz}\right)\;dC}{\theta_{t}}=\int_{C}\left(Gr\left(r\right)+2Gl_{t}^{2}\right)\;dC\\ \; & =G\left(\pi \frac{R_{o}^{4}-R_{i}^{4}}{2}\right)+G\pi 2l_{t}^{2}\left(R_{o}^{2}-R_{i}^{2}\right) \end{array}$$

with the corresponding rigidity ratio, $\Omega_{hco}$;

(33) $$\Omega_{hco}=\frac{J_{co}}{J_{ch}}=1+\frac{{\left(2l_{t}\right)}^{2}}{R_{o}^{2}+R_{i}^{2}}$$

By applying the same procedure in the case of solid cylinder, the following relation for the rigidity ratio is obtained, which coincides with the one proposed in [77]:

(34) $$\Omega_{sco}=\frac{J_{co}}{J_{cs}}=1+6{\left(\frac{l_{t}}{R}\right)}^{2}$$

## 4. Identification of Material Parameters and Discussion

To consider CNT as an equivalent micropolar or couple-stress non-local continuum model, one needs to know the corresponding non-local parameters as well as the nanotube thickness. In the present section, the torsional rigidities obtained from MD simulations in Section 2 and the continuum relations presented in Section 3 are incorporated through an optimisation approach to determine the non-local parameters and the tube thickness. The results obtained for the three continuum models, namely; micropolar, couple stress and classical Cauchy models for predicting the torsional rigidity of CNT are compared with each other, and with the results of MD simulations considered as benchmark solution.

For the optimisation approach we search for

(35) $$\min\{F_{obj}\left(G,N,\psi ,l_{t},h\right)\}\;$$

utilizing a non-linear least square method. $F_{obj}$ is a non-linear function which is the Euclidean norm of the difference between the torsional rigidity, “*J*”, directly obtained from the MD simulations and the ones predicted by micropolar model. As explained in Section 3, $J/I_{p}$ is equal to the apparent shear modulus, ΩG, in the continuum model;

(36) $$F_{obj}=\left|\frac{\frac{J_{\mathrm{MD}}}{I_{p}}-{\left(\Omega G\right)}_{\mathrm{Continuum}}}{\frac{J_{\mathrm{MD}}}{I_{p}}}\right|$$

For the micropolar case, the five unknown parameters are *G*, *N*, ψ, $l_{t}$ and the wall thickness, *h* (Equation (35)). Using the principle of non-negative energy, the first four unknowns are under the following inequality constraints [55,72]:

(37) $$0\leq G,\;0\leq N\leq 1,\;0\leq \psi \leq 1.5,\;0\leq l_{t}$$

Another inequality constraint is applied on the thickness based on the Vodenitcharova and Zhang criterion [78];

(38) 0<h≤1.42Å

The unknowns reduce to *G*, $l_{t}$ and *h* for the case of couple stress theory.

Note that, alternatively, the unknown variables could be considered as κ, α, β+γ, μ and *h*, with the following constraints instead of Equation (37) [73]:

(39) $$\begin{array}{c} 0\leq 2\mu +\kappa ,\;0\leq \kappa ,\;0\leq 3\alpha +\beta +\gamma \;0\leq \beta +\gamma \end{array}$$

However, once the four engineering non-local parameters are determined, the alternative non-local ones can be derived through the following relations;

(40) $$\kappa =\frac{2GN^{2}}{1-N^{2}},\;\alpha =\frac{2Gl_{t}^{2}}{\psi (1-\psi )},\;\beta +\gamma =2Gl_{t}^{2},\;\mu =\frac{2G-k}{2}$$

It should be noted that an optimisation problem is considered linear/non-linear if the objective function and/or the constraints are linear/non-linear and non-convex if the constraints define a non-convex problem. The existence and uniqueness of the solution can be guaranteed by Kuhn-Tucker conditions [79]. In the present problem, $F_{obj}$ is a non-linear function with the inequality constraints (Equation (37)) that define a non-convex domain. Although, in this case the existence and uniqueness of the solution is not guaranteed, with a proper selection of initial guesses a feasible and local minimum solution has been found for the problem.

By adopting the optimisation approach, the identified parameters for the micropolar model are presented in Table 2 for both armchair and zigzag CNTs;

In addition, Table 3 shows the obtained material parameters considering the couple stress theory.

Finally, in Table 4, the corresponding shear moduli for the classical Cauchy model are presented. Note that for Cauchy model, the only unknown parameter is the product of shear modulus and thickness, Gh, therefore, a freedom in the choice of thickness exist when considering the Cauchy model. In this regard, without the loss of generality, a thickness equal to the ones obtained in micropolar case are considered for the sake of comparison.

**Figure 4:**
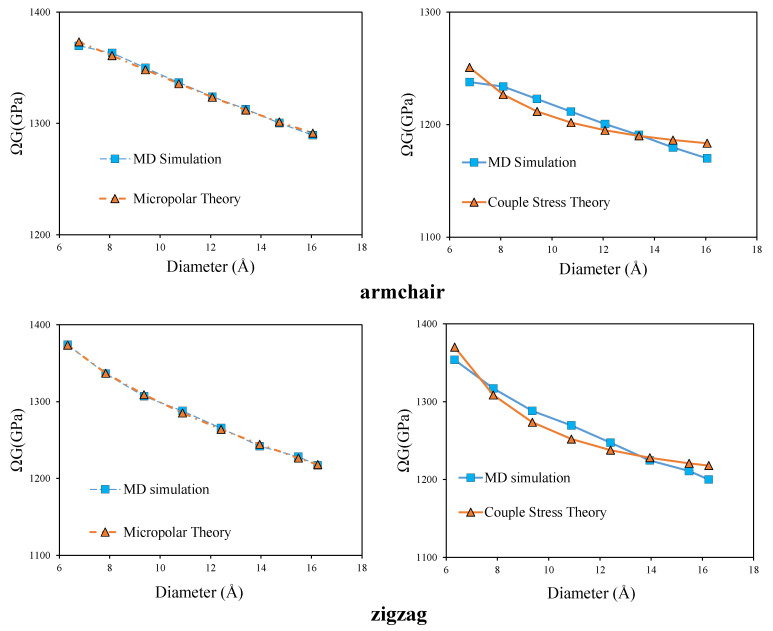
Comparison of apparent shear modulus from micropolar and couple stress models with MD results- the abscissa refers to the mean diameter, $d_{0}$.

It is clear from the Table 2 and Table 3, that the application of couple stress theory and Cauchy theory increases the norm of the objective function. For better clarification, in Figure 4 a comparison is made between the predicted apparent shear modulus by the micropolar and couple stress models with those obtained by MD simulation.

**Figure 5:**
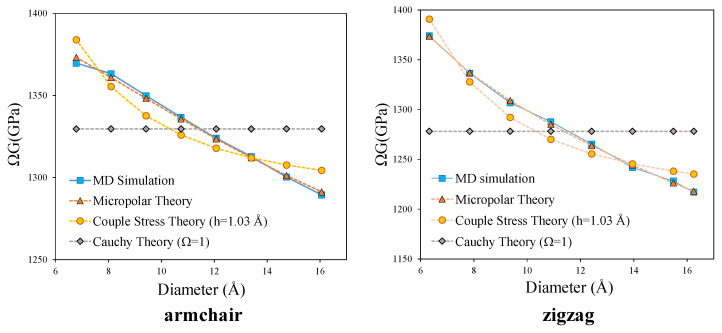
Comparison of apparent shear modulus from micropolar, couple stress and Cauchy models with MD results.

In addition, for a more general comparison, the prediction of apparent shear modulus by all the three theories considering the same wall thickness are presented in Figure 5. The occurrence of size effect in the torsional rigidity of armchair and zigzag CNTs is clearly captured in this figure. If the material could be modeled as a classical Cauchy material, (Ω=1), the shear modulus should have been constant for all the simulated CNTs, while, a decreasing trend is observed in MD simulation results. This decreasing trend can be predicted by both couple stress and micropolar theories. However, it is evident from the Figure 5 that the micropolar theory predicts the MD-simulated torsional rigidity much more successfully than the couple stress theory. Besides, the effective prediction of torsional behaviour by micropolar theory compared to couple stress theory, suggests the presence of non-symmetric stress and strain measures during the torsional loading of CNT, which can only be accounted for by micropolar theory. The presence of non-symmetric stress and strain measures, peculiarity of the micropolar model, may originate from the difference between the arrangements of carbon hexagonal lattices in the axial and circumferential directions of CNTs. It also indicates the existence of relative rotation (the difference between micro and macro rotation) which corresponds to the skew-symmetric part of stress and strain tensors.

In addition, to validate the obtained parameters, the product of $\Omega_{h}G\times h$ is compared with G×h in previously reported experimental and numerical results [80,81]. Gh is also known as surface modulus, however, in the present study $\Omega_{h}G$ is used instead of *G* to define the surface modulus. That is because in an experimental test or a simulation, the introduced apparent shear modulus is sensed as the rigidity modulus when a torque is applied on a single CNT (see Equation (25)). In an experiment conducted by Hall [81], a value of 410 GPa is reported for *G* considering a thickness of 3.4 Å for CNTs with diameters around 9.7 Å. This yields to a surface modulus of 139.4 GPa·nm. This is quite near to 139.3 GPa·nm value of surface modulus obtained in the present paper for SWCNT(7,7) with 9.4 Å diameter. Also, by performing MD simulations, Khoei et al. reported a value of 394 GPa with a thickness of 3.4 Å which results in a value of 134 GPa·nm for the surface modulus of a SWCNT(10,10) [80]. This value is also consistent with 135.5 GPa·nm for the surface modulus of SWCNT(10,10) in the present paper.

In order to show the transferability of the obtained micropolar parameters from the present approach, one can use the extracted values to predict other mechanical behavior of CNTs like critical torque. This can be a next step to be performed in a further study.

### 4.1. Equivalent Thickness

There is a controversy among researchers for the value of SWCNT’s thickness. The reported values for the effective thickness of SWCNTs have varied from 0.617 Å to 6.9 Å [64]. Khademolhosseini et al. [38] reported a value of 0.85 Å by matching critical torques obtained by MD-simulations and non-local shell model. Kudin et al. [82] used values of bending rigidity and in-plane stiffness obtained from ab-initio simulations together with their classical continuum definitions and reported a value of 0.89 for CNT shell thickness. One of the most important studies about the wall thickness of the SWCNTs was carried out by Vodenitcharova and Zhang [78]. Based on the consideration of force equilibrium and equivalence, they proposed a necessary condition (but not sufficient) for justifying an effective thickness of SWCNTs that should be smaller than the theoretical diameter of a carbon atom (∼1.42 Å). This is known as the Vodenitcharova-Zhang criterion. Their argument is that the cross-section of a nanotube contains only a number of atoms and that the forces in the nanotube are transmitted through these atoms. However, in a continuum mechanics-based model the same forces are transmitted through a continuous wall. Hence, the effective wall thickness cannot be greater than or equal to the theoretical diameter of a carbon atom, otherwise, the nanotube’s equilibrium cannot be maintained. Based on this criterion, the assumed value of 3.4 Å, as well as those larger than 1.42 Å, e.g., 6.9 Å and 1.47 Å, should be excluded in future investigations.

In the present work, the thickness is calculated through the optimisation process. It is found to be 1.033 Å and 1.035 Å, for armchair and zigzag CNTs, respectively. These values satisfies the Vodenitcharova and Zhang criterion and is near to the values reported in [38,82].

### 4.2. Internal Length Parameter

The internal length parameter is actually a measure of the internal structure of a material. It may refer to different physical features; the brick size in a masonry material, the size of reinforcing particles in a composite and the radius of gyration for a polymeric material are some of the examples. For nanostructures, the internal length parameter is considered to be in the order of atomic distances. Focusing on CNTs internal length parameter, $l_{t}$, some researchers used the fixed value of carbon-carbon bond (∼1.42 redÅ) in their non-local models [38]. Khajueenejad and Ghanbari [48] proposed different values for internal length parameter in the range of 4 to 10 Å for armchair and zigzag CNTs. Akbarzadeh and Soltani [49] added another length scale parameter to the modified couple stress theory named micro-inertia length scale parameter. The original length parameter varied while they fixed the so-called micro inertia parameter to be in the order of the lattice size.

**Figure 6:**
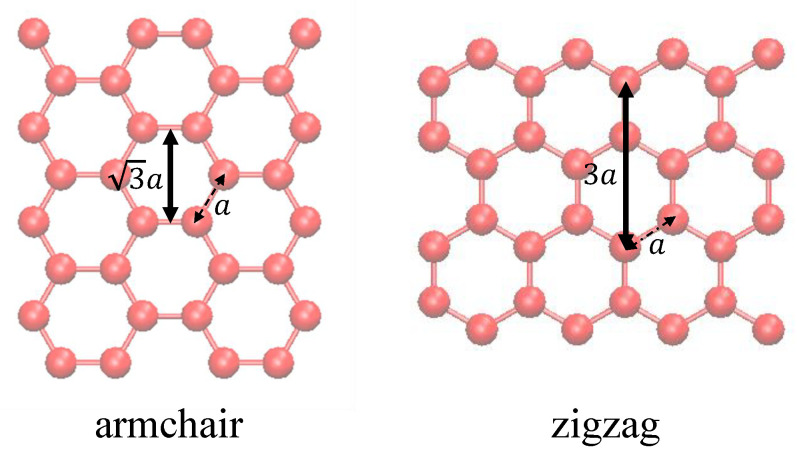
Periodic dimension in the axial direction related to the determined internal length parameter for zigzag and armchair CNTs, “a” is the carbon-carbon bond ∼1.42 Å.

In the present paper a unique internal length parameter is obtained from the optimisation procedure, for different diameters in each class of CNTs. This is more meaningful considering the lattice nature of the structure. The internal length parameter is determined as 2.3 Å and 3.7 Å for armchair and zigzag CNTs, respectively. These values can be related to the periodic dimension in the axial direction as illustrated in Figure 6. Also, from the above discussion, a reasonable prediction is that the value of $l_{t}$ for any chiral CNT should fall within the range of internal length of armchair and zigzag nanotubes as they form the extreme cases of chirality, this can be proved by analyzing a chiral CNT in a further study.

### 4.3. CNT as Solid Micropolar Cylinder

To complete this section, Table 5 presents the obtained material parameters considering CNT as a solid cylinder and adopting micropolar, couple stress and Cauchy continuum models with the same procedure previously described.

**Figure 7:**
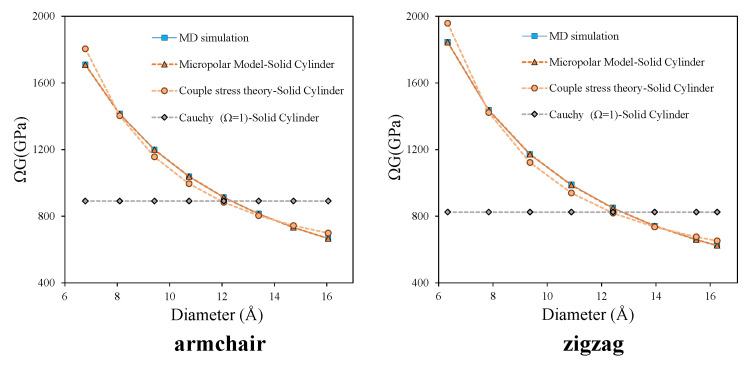
Comparison of apparent shear modulus from micropolar and couple stress and Cauchy models with MD results-solid cylinder assumption.

Considering the solid cylinder assumption, the comparison between the predicted apparent shear modulus by micropolar, couple stress and Cauchy models with those obtained by MD simulation are presented in Figure 7.

According to Table 5, the smallest objective norm still belongs to the micropolar model. Besides, Figure 7, clearly shows that the predicted values by micropolar theory matches very well with the MD-simulated results.

**Figure 8:**
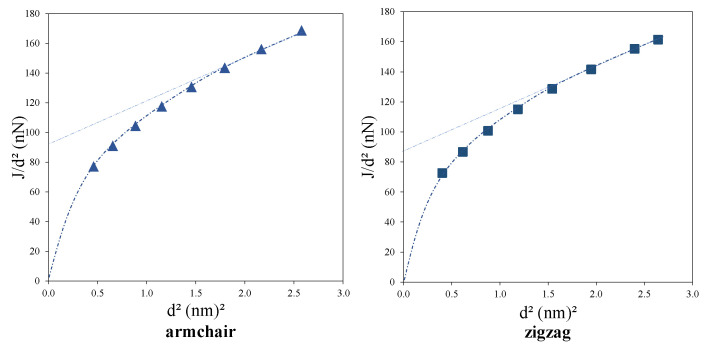
Plot of torsional rigidity divided by the square of the diameter vs. the square of the external diameter for armchair and zigzag CNTs.

The plot of $\frac{J}{d^{2}}$ vs. $d^{2}$ for armchair and zigzag CNTs is presented in Figure 8. The trend of obtained $\frac{J}{d^{2}}$ suitably obeys the micropolar one proposed by Lake in Figure 3. Passing a linear line through the last two data of each plot yields to the value of G=312 GPa and $l_{t}=3.5\;Å$ for armchair CNTs and G=257 GPa and $l_{t}=3.9\;Å$ for zigzag CNTs. Although the values predicted graphically by the Lake’s figure differ from the obtained values by the optimisation process, they are in the same orders. However, it seems that more scattered data for CNTs, especially considering larger diameters, are needed to increase the accuracy of the graphical prediction.

## 5. Conclusions

Common practise of carbon nanotubes (CNTs) in nanoscopic field reveals the need to determine corresponding mechanical properties. Although atomistic simulations provide accurate and deep information in that aspect, severe computational expenses are imposed on the problem which limits the size and time scale of the studied atomic system. Hence, when gross mechanical behaviour of a discrete assembly is looked for, it is favourable to resort equivalent non-classical continua formulations, which are capable of retaining the memory of internal material organisation while exploiting the efficiency of continuum analysis. The present paper aims to show the applicability of micropolar continuum theory, that is endowed with additional kinematic descriptors and work-conjugated skew-symmetric and couple stress measures, to model size dependent mechanical behaviour of CNTs, whose underlying lattice structure indicates the essence of micro-rotations. By comparing the apparent shear modulus of discrete models obtained from molecular dynamics simulation for moderately short nanotubes and the ones in continuum micropolar beam model, corresponding material properties as well as the tube thickness is derived, exploiting a non-linear optimisation procedure. The tube thickness is determined to be 1.033 Å and 1.035 Å, for armchair and zigzag CNTs, respectively which satisfies the Vodenitcharova and Zhang criterion and is consistent with reported values in literature. In addition, a unified internal length parameter is obtained for different diameters in each class of CNTs which is found to be relevant with their hexagonal lattice structure. Besides the common assumption of CNTs as hollow tubes, solid cross-sectional area is also considered as it can be expedient when dealing with a reinforcement element in composite structure. The predicted torsional rigidities are further compared with the ones obtained via couple stress and Cauchy theories. The results clearly depicts the success of micropolar theory as it is perfectly able to capture trend of discrete model by accounting not only for size effects, but also for non-symmetries in strain and stress measures in contrast to couple stress theory. In the present contribution, the torsional behaviour is considered due to a better correspondence between the boundary conditions of theoretical model and MD simulations, with which all the mechanical properties are obtained. However, the procedure can be further extended to different loading conditions with sensible scale-effects. The material parameters predicted in this study can also be used in different static problems of CNTs. Moreover, the number of MD simulations can be increased which will inherently improve the precision/accuracy of the optimisation procedure.

## Figures and Tables

**Table 1:** Parameters of carbon nanotubes (measured after energy minimization and thermal equilibrium steps) and corresponding results obtained from MD simulations.

	Chirality (m,n)	Diameter $d_{0}$ (Å)	Length *L* (Å)	Torsional Stiffness *K* (nN·nm)	Critical Angle $\theta_{\mathrm{crt}}$ (rad)
armchair	(5,5)	6.78	32.80	10.801	0.726
	(6,6)	8.09	40.06	14.877	0.600
	(7,7)	9.42	47.29	19.576	0.528
	(8,8)	10.74	54.56	24.863	0.468
	(9,9)	12.07	61.80	30.785	0.420
	(10,10)	13.40	66.61	38.678	0.396
	(11,11)	14.73	73.87	45.845	0.372
	(12,12)	16.06	81.12	53.613	0.354
zigzag	(8,0)	6.33	30.62	9.511	0.798
	(10,0)	7.84	39.03	13.662	0.654
	(12,0)	9.36	47.44	18.602	0.546
	(14,0)	10.89	53.02	25.716	0.432
	(16,0)	12.42	61.40	32.304	0.384
	(18,0)	13.95	68.38	40.312	0.336
	(20,0)	15.48	78.19	47.621	0.300
	(21,0)	16.25	82.38	51.759	0.288

**Table 2:** Identified material parameters for armchair and zigzag CNTs as micropolar materials.

Parameter	G	$l_{t}$	ψ	N	*h*	α	β+γ	μ	κ	norm ($F_{\mathrm{obj}}$)
Unit	GPa	Å	-	-	Å	nN	nN	GPa	GPa	-
Armchair	1177	2.3	1.5	0.15	1.032	−43	129	1152	51	0.0039
Zigzag	1016	3.7	1.49	0.19	1.035	−94	285	979	74	0.0039

**Table 3:** Identified material parameters for armchair and zigzag CNTs as couple stress materials.

Parameter	G	$l_{t}$	*h*	norm ($F_{\mathrm{obj}}$)
Unit	GPa	Å	Å	-
Armchair	1168	0.6	1.14	0.02
Zigzag	1190	0.9	1.05	0.03

**Table 4:** Identified material parameters for armchair and zigzag CNTs as classical materials.

Parameter	G	*h*	norm ($F_{obj}$)
Unit	GPa	Å	-
Armchair	1330	1.032	0.06
Zigzag	1278	1.035	0.11

**Table 5:** Identified material parameters for armchair and zigzag CNTs as solid cylinders.

Parameter	G	$l_{t}$	ψ	N	norm ($F_{\mathrm{obj}}$)
Unit	GPa	Å	-	-	-
Micropolar					
Armchair	214	5.5	1.4	0.7	0.0022
Zigzag	184	6	1.4	0.7	0.0043
Couple stress					
Armchair	459	2.4	-	-	0.01
Zigzag	418	2.5	-	-	0.012
Cauchy					
Armchair	891	-	-	-	0.78
Zigzag	825	-	-	-	0.78

## Data Availability

The data presented in this study are available on request from the corresponding author.
